# Case of Hæmorrhage of the Liver

**Published:** 1844-08-24

**Authors:** J. Abercrombie

**Affiliations:** The Cape of Good Hope


### CASE OF HÆMORRHAGE OF THE LIVER.

By J. ABERCROMBIE, M.D. of the Cape of Good Hope.

The patient was a lady, 35 years of age, who, during the last two months of her pregnancy, had suffered much from dyspepsia. On the 28th September, 1841, she was seized with severe pain in the epigastrium, a sense of distention, eructations, and nausea. Having on former occasions been relieved of (what she considered) a similar pain by pressure, she had recourse to it on this occasion, by bandaging the waist, and to such an extent, as to cause the author to fear mischief might be produced. Opium and ether were given, and in the evening she was quite relieved. On the following morning, however, labour came on; it was of a short duration; the placenta was readily expelled, and the uterus contracted well. Within an hour, however, symptoms of sinking appeared, leading the author to suspect uterine hæmorrhage, but on examination the uterus was found perfectly contracted, and the discharge outwardly very moderate. She complained of pain in the right hypochondrium, and right side of the neck. Opium and stimulants were freely given, and for a time she seemed to rally, but vomiting supervened; collapse of the system took place, and she died on the morning of the 1st of October. On examination after death, there was found on the anterior and inferior surfaces of the liver a large sac, which burst on attempting to remove the organ, and discharged about two pounds of blood, fluid and coagulated. It was found to have escaped from a branch of the vena porta, and the sac was formed by the peritoneum. The organ itself had throughout a mottled apearance, and was unusually soft. The uterus was in a perfectly sound state, as were all the other organs of the viscera, both of the pelvis and abdomen.—*London Med. Times.*

